# Astragaloside IV Attenuates Glutamate-Induced Neurotoxicity in PC12 Cells through Raf-MEK-ERK Pathway

**DOI:** 10.1371/journal.pone.0126603

**Published:** 2015-05-11

**Authors:** Rongcai Yue, Xia Li, Bingyang Chen, Jing Zhao, Weiwei He, Hu Yuan, Xing Yuan, Na Gao, Guozhen Wu, Huizi Jin, Lei Shan, Weidong Zhang

**Affiliations:** 1 School of Pharmacy, Second Military Medical University, Shanghai, China; 2 School of Pharmacy, Shanghai Jiao Tong University, Shanghai, China; 3 Department of Mathematics, Logistical Engineering University, Chongqing, China; 4 School of Pharmacy, East China University of Science and Technology, Shanghai, China; China Medical University, TAIWAN

## Abstract

Astragaloside IV (AGS-IV) is a main active ingredient of *Astragalus membranaceus* Bunge, a medicinal herb prescribed as an immunostimulant, hepatoprotective, antiperspirant, a diuretic or a tonic as documented in Chinese Materia Medica. In the present study, we employed a high-throughput comparative proteomic approach based on 2D-nano-LC-MS/MS to investigate the possible mechanism of action involved in the neuroprotective effect of AGS-IV against glutamate-induced neurotoxicity in PC12 cells. Differential proteins were identified, among which 13 proteins survived the stringent filter criteria and were further included for functional discussion. Two proteins (vimentin and Gap43) were randomly selected, and their expression levels were further confirmed by western blots analysis. The results matched well with those of proteomics. Furthermore, network analysis of protein-protein interactions (PPI) and pathways enrichment with AGS-IV associated proteins were carried out to illustrate its underlying molecular mechanism. Proteins associated with signal transduction, immune system, signaling molecules and interaction, and energy metabolism play important roles in neuroprotective effect of AGS-IV and Raf-MEK-ERK pathway was involved in the neuroprotective effect of AGS-IV against glutamate-induced neurotoxicity in PC12 cells. This study demonstrates that comparative proteomics based on shotgun approach is a valuable tool for molecular mechanism studies, since it allows the simultaneously evaluate the global proteins alterations.

## Introduction

*Astragali Radix*, one of the most commonly used traditional Chinese medicine (TCM), is prepared from the roots of *Astragalus membranaceus* (Fisch) Bunge and *Astragalus mongholicus* Bunge, and is prescribed as an immunostimulant, hepatoprotective, antiperspirant, a diuretic or a tonic as documented in Chinese Materia Medica [1]. A brief survey of the therapeutic functions of *Astragali Radix* includes immunoregulatory, antioxidant, anti-cancer, antiviral, diuretic, hypolipidemic, and hypoglycemic effects, as well as its protective effects toward cardiovascular, liver, lung, and neural tissues as well as upon renal function [2–5]. Total *Astragalus membranaceus* extract, mainly composed of astragalosides and astragalus polysaccharides, is often employed in different *in vitro* and *in vivo* studies without significant toxic effects. Regarding the chemical constituents of *Astragali Radix*, more than 100 compounds, such as isoflavonoids, triterpene saponins, polysaccharides and amino acids, have been identified so far, and various biological activities of the compounds have been reported [6]. Astragalosides are cycloartane triterpenoid saponins which are characterized with 3-, 6- and/or 25- conjugated glucose moieties and whose 3-glucose is acetylated [7]. The basic aglycone form of these triterpenes is known as cycloastragenol (9,10-cyclo-lanostane type; CAG) and a 3,6-glycosylated relative without acetylation named astragaloside IV (3-0-beta-d-xylopyranosyl-6-0-beta-d-glucopyranos-ylcycloastragenol, AGS-IV), which is more abundant in *Astragali membranaceus* than in other *Astragalus* species [7]. AGS-IV, one of the major and active components of the *Astragalus membranaceus*, has been renowned for a series of protective effects, such as anti-oxidant [8–10], anti-hypertension [11], anti-diabetic [12], angiogenic [13], anti-infarction [14–17], anti-inflammation [18], healing and anti-scar effects [19].

Our recent studies have shown that AGS-IV can show cardioprotection during myocardial ischemia *in vivo* and *in vitro* [20]. AGS-IV dilates aortic vessels from normal and spontaneously hypertensive rats through endothelium-dependent and endothelium-independent ways [21]. Moreover, we have demonstrated that AGS-IV inhibited vessel contraction through blocking calcium influx and intracellular calcium release. The endothelium-dependent vessel dilation of AGS-IV is attributed mainly to the endothelium-dependent nitric oxide (NO)-cGMP pathway [22].

Although AGS-IV can exert cardioprotection activity under pathophysiological conditions, the targets of AGS-IV characterization is a bottleneck and its mechanism remains to be elucidated. Recently, we have investigated the therapeutic mechanism of AGS-IV against cardiovascular diseases using a network-based methodology that integrates data of drugs, targets and pathways [16]. However, the detailed molecular bases of protective effects of AGS-IV on the proteome level remain largely unexplored.

Proteins are central to the understanding of cellular function and disease processes. Proteomics in general deals with the large-scale determination of gene and cellular function directly at the proteome level, and the MS-based proteomics has established itself as an indispensable technology to interpret the information encoded in proteome [23–25]. The proteomic approach is widely applied nowadays in the development of novel biomarker candidates for early detection of disease and identification of new targets for therapeutics, mainly by delineation of protein expression changes depending on factors such as the organism’s physiological state and the stage of development of disease [26].

In the present study, we employed a high-throughput comparative proteomic approach based on 2D-nano-LC-MS/MS to investigate the possible signaling pathways involved in the neuroprotective effect of AGS-IV against glutamate-induced neurotoxicity in PC12 cells. The main purpose of the present study was to explore the detailed molecular bases of the neuroprotective effect of AGS-IV on the proteome level with comparative proteomics, so as to get better knowledge of neuroprotective mechanism of AGS-IV.

## Materials and Methods

### Chemicals

**Fig 1:**
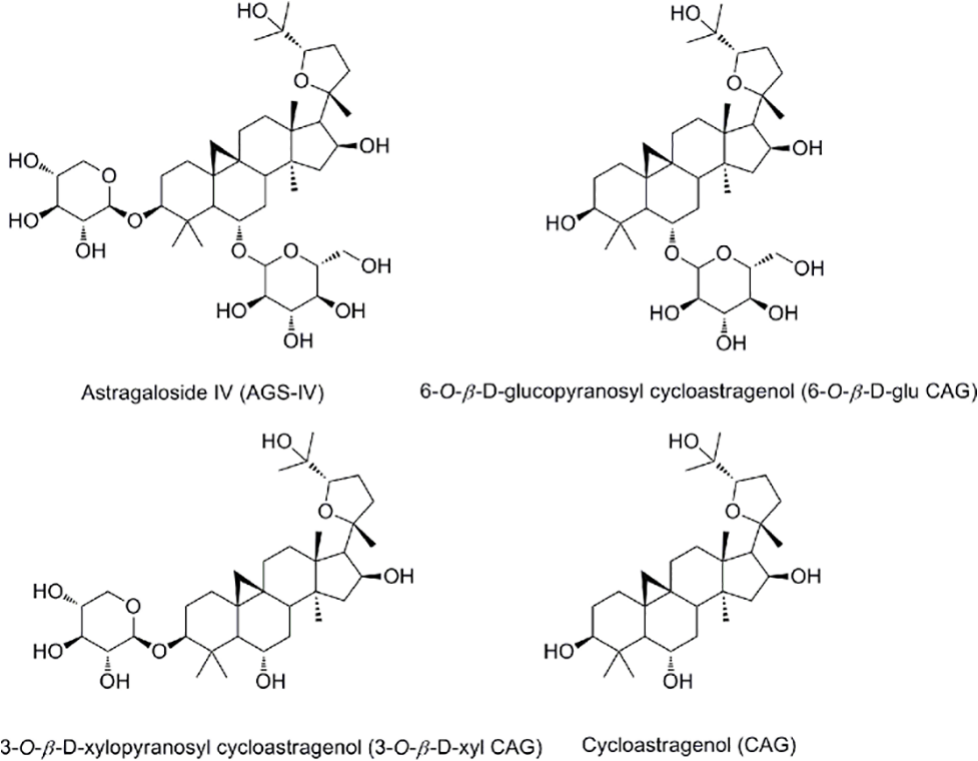
Chemical structures of the four compounds, astragaloside IV (AGS-IV), 6-*O*-*β*-*D*-glucopyranosyl cycloastragenol (6-*O*-*β*-*D*-glu CAG), 3-*O*-*β*-*D*-xylopyranosyl cycloastragenol (3-*O*-*β*-*D*-xyl CAG), cycloastragenol (CAG).

CAG, 6-*O*-*β*-*D*-glucopyranosyl cycloastragenol (6-*O*-*β*-*D*-glu CAG), 3-*O*-*β*-*D*-xylopyranosyl cycloastragenol (3-*O*-*β*-*D*-xyl CAG), and AGS-IV were isolated from the dried root tuber of *Astragalus membranaceus* and identified by ^1^H NMR, ^13^C NMR, ESI-MS data and by comparing with the published data of earlier studies [27] (purity > 98% as determined by HPLC). Their chemical structures are shown in **Fig 1**.

### Reagents

Dimethyl sulfoxide (DMSO) and [3-(4,5-dimethylthiazol-2-yl)-2,5-diphenyltetrazolium bromide] (MTT) was purchased from Sigma (St Louis, MO, USA). Dulbecco’s Modified Eagle’s Medium (DMEM), fetal bovine serum (FBS) were purchased from Gibco (Grand Island, NY). Antibodies for p-Raf, p-MEK, p-ERK1/2, ERK1/2, p-JNK, JNK, and β-actin were obtained from Cell Signaling Technology (Beverly, MA). Vimentin and Gap43 were purchased from Santa Cruz Biotechnology (Santa Cruz, CA, USA). HPLC-grade acetonitrile was obtained from Merck KGaA (Darmstadt, German).

### Cells and drug treatments

The high differentiated rat pheochromocytoma tumor cell line PC12, obtained from the Cell Bank of the Shanghai Institute of Biochemistry & Cell Biology, Shanghai Institute for Biological Sciences, Chinese Academy of Sciences (Shanghai, China, http://www.cellbank.org.cn), was maintained in DMEM containing 10% FBS supplemented with 100 U/mL penicillin and 100 μg/mL streptomycin in a humidified atmosphere containing 5% CO_2_ at 37°C as described previously [28]. AGS-IV was dissolved in DMSO and was freshly prepared each time before use (DMSO final culture concentration < 0.1%). For cell cytotoxicity assays, PC12 cells were inoculated at a density of 1 × 10^4^ cells per well in 96-well microplates and then maintained for a 30-h incubation with test compounds. For neuroprotective assay, PC12 cells were treated with test compounds for 6 h before exposure to 5 mM glutamate and then maintained for 24 h.

### MTT and LDH release assay

The effects of the test compounds on the cytotoxicity were determined by the MTT and LDH release assay as described previously [28]. Briefly, PC12 cells were seeded in 96-well plates at 1 × 10^4^ cells per well and co-incubated with the test compounds for 24 h. Cultures were also treated with 0.1% DMSO as the untreated control. After treatment, 10 μL of MTT solution (5 mg/mL) was added to each well and the plates were incubated for 4 h at 37°C. The supernatant was then removed from formazan crystals and 100 μL of DMSO was added to each well. The absorbance at 570 nm was recorded using a SYNERGY Mx microplate reader (BioTek, Winooski, VT). The cell survival percentages were calculated by dividing the mean OD of compound-containing wells by that of control wells. LDH assay was performed by a commercial kit (Promega, Madison, WI, USA) according to the manufacturer’s protocol. PC12 cells were treated with a range of compounds concentrations for 24 h. Lysis solution was added to 96-well plates and incubated at 37°C for 30 min. Then supernatant of each well was moved to a new 96 well plate and reconstituted substrate was added and the plates were kept for another 30 min in dark at room temperature. Stop solution was then added to each well and left for 30 min. Absorbance was recorded at 490 nm. The percentage release of LDH from the treated cells was calculated by comparing it to the maximum release of LDH achieved by the lysis solution used on the control cells.

### Trypan blue viability assay

PC12 cells (5 × 105) were seeded in six-well plates exposed to 5 mM glutamate and 25, 50, and 100 μM AGS-IV. After 24 h, the cells were stained with trypan blue (Bio-Rad) and the amount of living cells was determined using an automat Cell Counter (Bio-Rad).

### Apoptosis assay

Cells in early and late stages of apoptosis were detected using an Annexin V-FITC apoptosis detection kit from BD Biosciences. In this procedure, 5 × 10^5^ PC12 cells per well in six-well plate were exposed to glutamate and/or test compounds and incubated for 24 h prior to analysis. Cells were collected and washed with phosphate buffer (PBS), then re-suspended in Annexin V binding buffer followed by addition of Annexin V-FITC and propidium iodide (PI). The samples were incubated in the dark for 5 min at room temperature and analyzed with a FACSCaliber flow cytometer (BD Biosciences, San Jose, CA). Mean of % apoptosis index was calculated by combining early apoptosis (annexin V^+^/PI^+^) and late apoptosis (annexin V+/PI-) events.

### LC-MS Sample preparation

Different processing PC12 cells were homogenized in lysis buffer (8 M urea, 50 mM Tris-HCl, pH 7.5, 0.25% v/v Triton X-100, 1 mM phenylmethylsulfonyl fluoride (PMSF), 1 mM dithiothreitol, 1 × protease inhibitor cocktail) and centrifuged at 12,000 rpm for 10 min at 4°C, and the supernatant was mixed with five volumes of precipitation buffer (ethanol: acetone: glacial acetic acid = 10: 10: 0.1). After washed three times with cold acetone, the pellet was dissolved in denature buffer (8 M urea, 50 mM Tris-HCl, pH 8.2). The sample was reduced by dithiothreitol at 37°C for 2 h and alkylated by iodoacetamide in the dark at room temperature for 30 min. Then the solution was diluted to 1 M urea with 50 mM Tris-HCl (pH 8.2). Finally, trypsin was added at an enzyme-to-substrate of 1/25 (w/w) and incubated at 37°C overnight. The digested mixture was loaded on a homemade C18 solid-phase cartridge (20 mg, 1 mL) conditioned with 0.5 mL × 3 acetonitrile and 0.5 mL × 3 water (containing 0.1% trifluoroacetic acid), then the cartridge was washed with 0.5 mL × 3 water (containing 0.1% trifluoroacetic acid) and eluted with 0.5 mL × 2 80% acetonitrile (containing 0.1% trifluoroacetic acid). The elution was speedvaced to dry and stored at -80°C before LC-MS analysis.

### 2D-nano-LC-MS/MS analysis and database searching

The 2D-nano-LC-MS/MS system applied a quaternary surveyor pump and an LTQ linear ion trap mass spectrometer equipped with a nanospray source (Thermo, San Jose, CA). The tryptic samples were dissolved in 0.1% formic acid, loaded onto a monolith strong cation exchange (SCX) column (150 μm id × 7 cm) automatically. Then a series stepwise elution with salt concentrations of 50, 100, 200, 300, 400, 500, and 1000 mM NH_4_AC were used to gradually elute peptides from the phosphate monolithic column onto the C18 analytical column. Each salt step lasts 10 min except for the last one, which lasts for 20 min after the whole system was re-equilibrated for 10 min with buffer A (0.1% formic acid water solution), the binary gradient elution with buffer A and buffer B (0.1% formic acid acetonitrile solution) (0–10 min, from 0 to 10% buffer B, 10–60 min, from 10 to 40% buffer B, 60–65 min, from 40 to 80% buffer B, 65–75 min, 80% buffer B, 75–85 min, buffer A) for reversed phase separation was applied prior to MS detection in each cycle. The temperature of the ion transfer capillary was set at 150°C. The spray voltage was set at 2.2 kV. All MS and MS/MS spectra were acquired in the data-dependent mode. The mass spectrometer was set so that one full MS scan was followed by three MS/MS scans. The MS/MS spectra were searched using Mascot against a composite database including both original and reversed rat protein database of International Protein Index (IPI_RAT RAT_v3.26). Peptides were searched using fully tryptic cleavage constraints and up to two missed cleavages sites were allowed for tryptic digestion. The mass tolerances are 2 Da for parent masses and 1 Da for fragment masses. Initial searching results were filtered with the following parameters as tasted previously: the Xcorr g 1.8 for a singly charged peptide, 2.5 for a doubly charged peptide, and 3.5 for a triply charged peptides; the minimum ∆Cn cutoff value of 0.08. For semiquantitative comparisons of the proteins identified, spectral counts for each identified protein from each experiment were extracted, averaged, and compared as described previously [29–31].

### Data processing and principle component analysis (PCA)

The processed data of the comparative proteomics were exported and further processed by PCA using the SIMCA-P software package (Version 11, Umetrics, Umea, Sweden). The data were processed by unit variance scaling and were mean-centered using the SIMCA-P software.

### Network construction and Identification of pathways enriched with AGS-IV associated proteins

Network construction was performed as previously described [16]. The protein-protein interaction network was constructed based on the Human Protein Reference Database (HPRD) data and mapped all putative targets of AGS-IV onto this network. Then we applied the Steiner minimal tree algorithm to identify a minimum sub-network [32], which included as many putative targets of AGS-IV and as few other proteins as possible. Each target protein of AGS-IV was allowed to interact with other target proteins through no more than one non-target protein.

Differentially expressed proteins in the experiments were considered AGS-IV associated genes. These genes were mapped onto the pathways in the KEGG database, and then P-values were used to determine whether a pathway was enriched with AGS-IV associated genes instead of by chance. Assuming that the total AGS-IV associated genes (*K*) were mapped onto the rat pathways in KEGG, which included *N* distinct genes, a hypergeometric cumulative distribution function could model the probability of identifying at least *k* genes from a pathway of size *n* by chance, such that the P-value is given by:

$$P=1-\sum_{i=0}^{k-1}f(i)=1-\sum_{i=0}^{k-1}\frac{\left(\begin{array}{c} K\\ i \end{array}\right)\left(\begin{array}{c} N-K\\ n-i \end{array}\right)}{\left(\begin{array}{c} N\\ n \end{array}\right)}$$

Given the significance level *α* = 0.01, a P-value smaller than *α* demonstrated a low probability that the AGS-IV associated genes appeared in the pathway by chance; i.e., the pathway can be regarded as being significantly regulated by these gene-encoded proteins.

### Western blotting study

Serial concentrations of compound were added to 1×10^6^ PC12 cells in six-well plate. After 24 h of incubation at 37°C, the cells were collected by centrifugation at 1000 rmp for 5 min. The cell pellets were then washed with PBS, resuspended in lysis buffer containing 150 mM NaCl, 50 mM Tris (pH 8.0), 0.02% NaN_3_, 0.01% PMSF, 0.2% Aprotinin, 1% TritonX-100 supplemented with protease inhibitor cocktail (Sigma. St. Louis, MO), and centrifuged at 12 000 rmp for 10 min. The concentration of total proteins was determined using a BCA kit (Pierce, Rockford, IL). 40 μg protein per lane was electrophoresed on 12% SDS polyacrylamide gels after boiling for 5 min and transferred to polyvinylidene difluoride (PVDF) membrane. Nonspecific reactivity was blocked by 5% nonfat milk prepared in TBST (10 mM Tris, 150 mM NaCl, 0.05% Tween-20, pH 7.5) at room temperature for 1 h. The membranes were incubated with antibodies diluted according to the manufacturers' instructions. The image was captured by the Odyssey infrared imaging system (Li-Cor Bioscience, Lincoln, NE). Protein densitometry was done using Quantity One imaging software (Bio-Rad) and normalized against β-actin.

### Statistical analysis

Data were analyzed using GraphPad Prism software version 5 (Graph Pad software Inc., San Diego, CA, USA). The comparison between two groups was analyzed by unpaired Student *t*-test, and multiple comparisons were compared by one-way ANOVA analysis of variance followed by Tukey post hoc test. The quantitative data were reported as means ± SEM from at least three independent experiments. Statistical significance was determined as P < 0.05.

## Results

### AGS-IV protects against glutamate-induced neurotoxicity in PC12 cells

**Fig 2:**
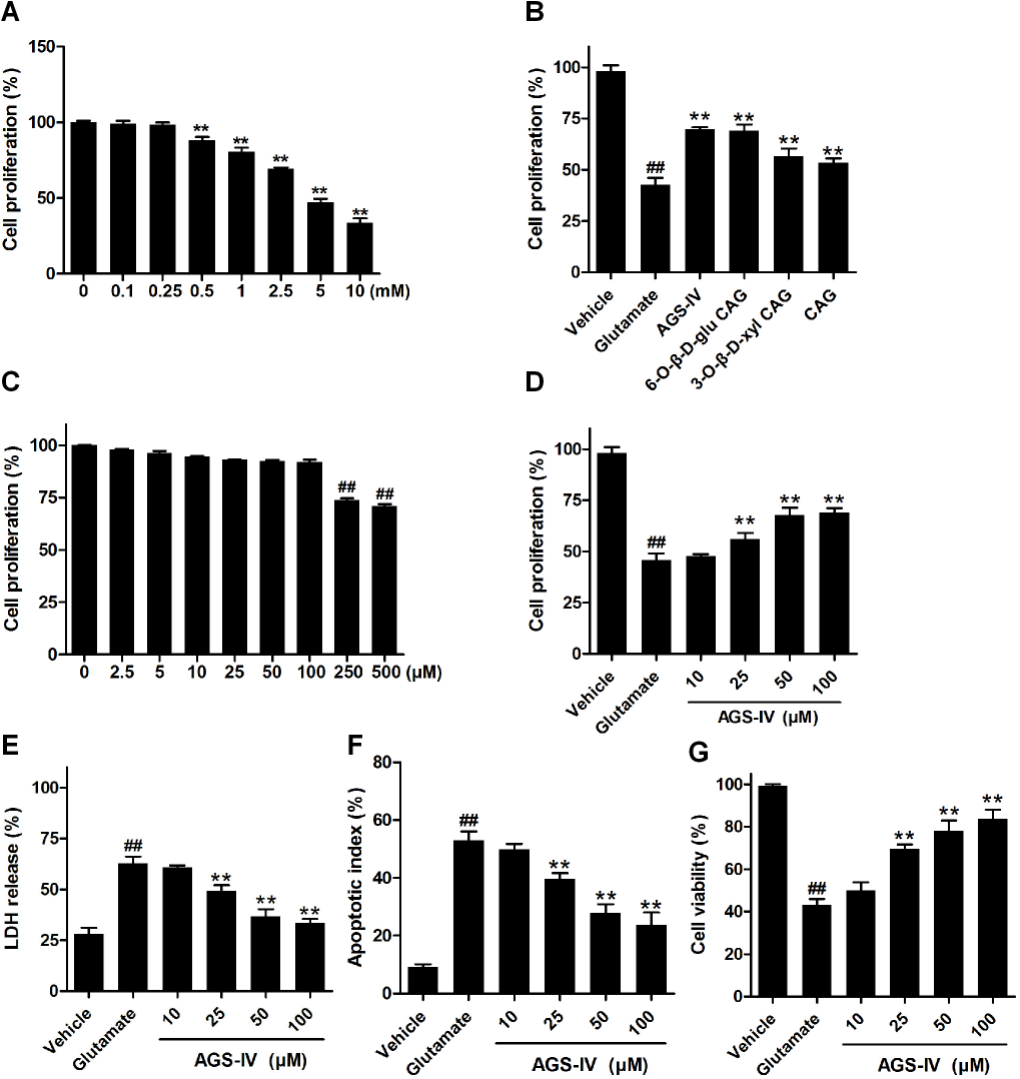
AGS-IV protects against glutamate-induced neurotoxicity in PC12 cells. (A) Dose-dependent cell death was observed after the cells were treated with different concentrations glutamate for 24 h. (B) Effects of 50 μM AGS-IV, 6-*O*-*β*-*D*-glu CAG, 3-*O*-*β*-*D*-xyl CAG, and CAG on glutamate-induced PC12 cell neurotoxicity. (C) Survival of PC12 cells after exposure to 0.1% DMSO or various concentrations of AGS-IV for 30 h. (D, E) Effects of AGS-IV on glutamate-induced PC12 cell injury. PC12 cells were treated with 10, 25, 50 and 100 μM AGS-IV and then co-incubated with or without 5 mM glutamate for 24 h, and cell cytotoxicity was determined by MTT assay and LDH activity. (F) Apoptosis assay of PC12 cells exposed to glutamate and/or AGS-IV were examined by flow cytometry. PC12 cells were gated for an annexin V^+^ (x-axis) versus PI^+^ (y-axis) contour plot. The numbers on dot plots represent the percentages of annexin V^+^/PI^+^ cells and annexin V^+^/PI^-^ cells. (G) The percentage of living cells was tested by trypan blue exclusion for PC12 cells exposed to glutamate and/or AGS-IV. All the data are presented as mean ± SEM of three independent experiments. ## < 0.01 versus vehicle and **P < 0.01 versus glutamate by one-way ANOVA analysis of variance with Tukey’s HSD post hoc test.

We evaluated whether the four cycloartane triterpenoid saponins extracted from the root tubers of *Astragalus membranaceus* could protect against oxidative glutamate cytotoxicity in vitro. As shown in **Fig 2A**, a clear dose-dependent cell death was observed after the cells were treated with different concentrations glutamate for 24 h. Thus, 5 mM was the preferred concentration of choice for the rest of the experiments. Examination of cytotoxicity of PC12 cells by the MTT method showed that 5 mM glutamate noticeably reduced the survival of PC12 cells with a rate of 43 ± 3.4% (P < 0.05 vs. vehicle) (**Fig 2B**). Among the saponins examined at 50 μM, AGS-IV and 6-*O*-*β*-*D*-glu CAG showed better protective effects than 3-*O*-*β*-*D*-xyl CAG and CAG (**Fig 2B**). Interestingly, cytotoxicity analysis showed AGS-IV at the concentrations exceeding 100 μM was found to be low cytotoxic to PC12 cells (**Fig 2C**). We further characterized the effect of AGS-IV (a major active ingredient, higher yield than that of the other three compounds) on glutamate cytotoxicity over a wide range of concentrations from 10 to 100 μM and found that AGS-IV could dose-dependently mitigate glutamate-induced neurotoxicity (**Fig 2D**). Similar findings were also obtained with LDH release assays with the rate of LDH release at 63 ± 3.5% in PC12 cells exposed to 5 mM glutamate and 49 ± 3.0%, 37 ± 3.6%, and 33 ± 2.1%, respectively, in PC12 cells exposed to 5 mM glutamate and 25, 50, and 100 μM AGS-IV (P < 0.01 vs. glumatate) (**Fig 2E**). In addition, flow cytometric analysis showed that glutamate induced apoptotic death of PC12 cells and AGS-IV (25, 50 and 100 μM) attenuated these apoptotic changes (**Fig 2F**). Moreover, 5 mM glutamate displayed a high toxicity in the trypan blue staining as in the MTT assay and AGS-IV could dose-dependently mitigate glutamate-induced neurotoxicity (**Fig 2G**).

### AGS-IV induces broad changes in protein expression in PC12 cells

**Fig 3:**
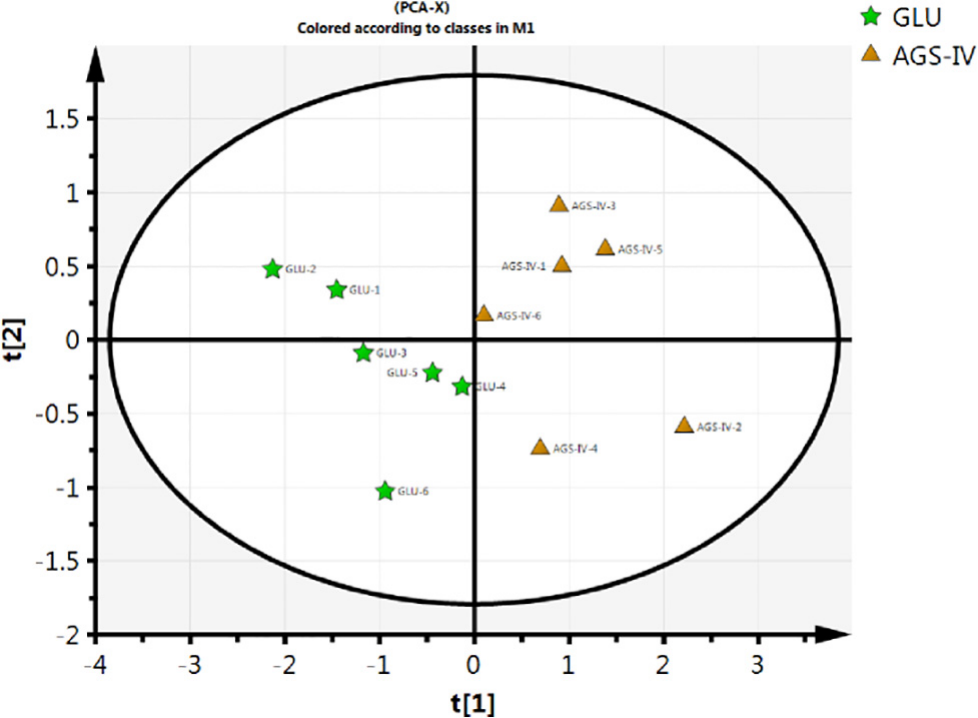
Score plot of PCA performed on the spectral count data of glutamate-treated groups (GLUs) and AGS-IV-treated groups (AGS-IVs) (Pentagram, GLUs; Triangle, AGS-IVs). Proteins meet the following filter criteria were included for PCA: (1) the number of unique peptide identified more than 2; (2) protein identified at least four out of six samples; (3) statistical significance (P < 0.05) were obtained by Student’s t-test (GLUs and AGS-IVs).

**Fig 4:**
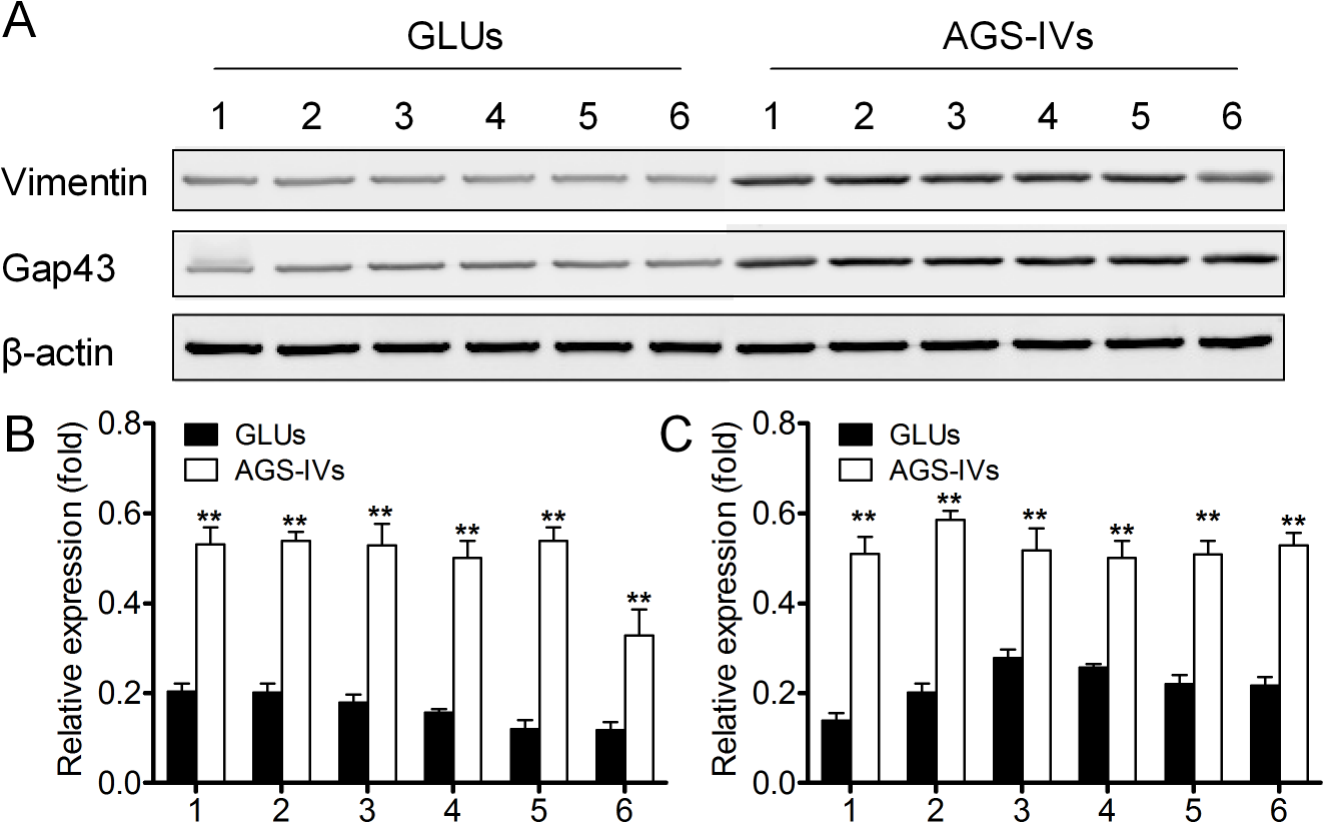
Western blotting of vimentin and Gap43 expression in GLUs and AGS-IVs. (A) Modulation of vimentin and Gap43 by AGS-IV on glutamate-induced PC12 cell injury. Data shown are the results of three different experiments and are represented as the relative densities of vimentin (B) and Gap43 (C) protein bands normalized to β-actin. Results are presented as means ± SEM of three assays. ** Significant difference compared with GLUs by Student’s t-test (P < 0.01).

A comprehensive shotgun proteomic profiling procedure, based on online 2D-nano-LC-MS/MS system was applied to uncover proteomic alterations associated with 100 μM AGS-IV treated PC12 cells exposed to 5 mM glutamate at 24 h. The numbers of total spectral counts and identified proteins are listed in **Table 1**. For PCA of the proteins identified in the striatum of AGS-IV treated PC12 cells (AGS-IVs) and 5 mM glutamate treated PC12 cells (GLUs), spectral counts for each identified protein from each experiment were extracted, averaged, normalized. To improve the reliability of identification of the proteins, proteins meet the stringent filter criteria (the number of unique peptide identified more than 2; protein identified at least four out of six samples) were included for the further Student’s t-test statistic. A total of 72 proteins were selected based on the filter criteria and a Student’s t-test statistic (p < 0.05). PCA was performed on this data set, which reveals two distinct clusters corresponding to AGS-IVs and GLUs (**Fig 3**). The proteins (listed in **Table 2**) meet the following filter criteria: (a) for the average spectral of AGS-IVs and GLUs, the larger value more than 4; (b) the spectral count ratio more than 3-fold and no more than 10-fold were included for the further functional discussion [33]. Two proteins, vimentin and Gap43, were randomly selected from whose antibodies are commercially available and their expression levels were further confirmed by Western Blots. **Fig 4** shows that the altered intensity of the proteins matched well with the differences obtained in 2D-nano-LC-MS/MS based proteomic analysis.

**Table 1:** Total spectral counts and proteins identified of glutamate groups (GLUs)^a^ and AGS-IV groups (AGS-IVs)^b^.

	GLUs						AGS-IVs					
	GLU-1	GLU-2	GLU-3	GLU-4	GLU-5	GLU-6	AGS-IV-1	AGS-IV-2	AGS-IV-3	AGS-IV-4	AGS-IV-5	AGS-IV-6
Total spectral counts	30458	30123	36893	39785	38699	39952	40088	46583	39536	41503	41368	39329
Total protein groups	1738	1315	1612	1849	1871	1420	2602	2726	2317	2023	2711	2107

a glutamate groups (GLUs): PC12 cells received 5 mM glutamate and then maintained for 24 h.

b AGS-IV groups (AGS-IVs): PC12 cells were treated with 50 μM AGS-IV for 6 h before exposure to 5 mM glutamate and then maintained for 24 h

**Table 2:** Lists of the identified differentially expressed proteins.

No.	Protein name	Ratio ^a^
1	Malate dehydrogenase	0.22
2	Heat shock protein HSP 90-alpha	0.33
3	14-3-3 protein epsilon	0.31
4	14-3-3 protein zeta/delta	0.24
5	Gap43	0.23
6	Neutral ceramidase	3.7
7	Heat shock 70 kDa protein 1A/1B	0.24
8	Serine/threonine-protein kinase TAO3	0.23
9	Myosin-9	0.31
10	Ribosome-binding protein 1	3.2
11	Vimentin	0.29
12	ribosomal protein S7	0.33
13	Periaxin	0.21

a Ratio averaged glutamate groups (GLUs) to AGS-IV groups (AGS-IVs) spectral counts.

### Protein-protein association network construction, pathways enriched and validation with AGS-IV associated proteins

**Fig 5:**
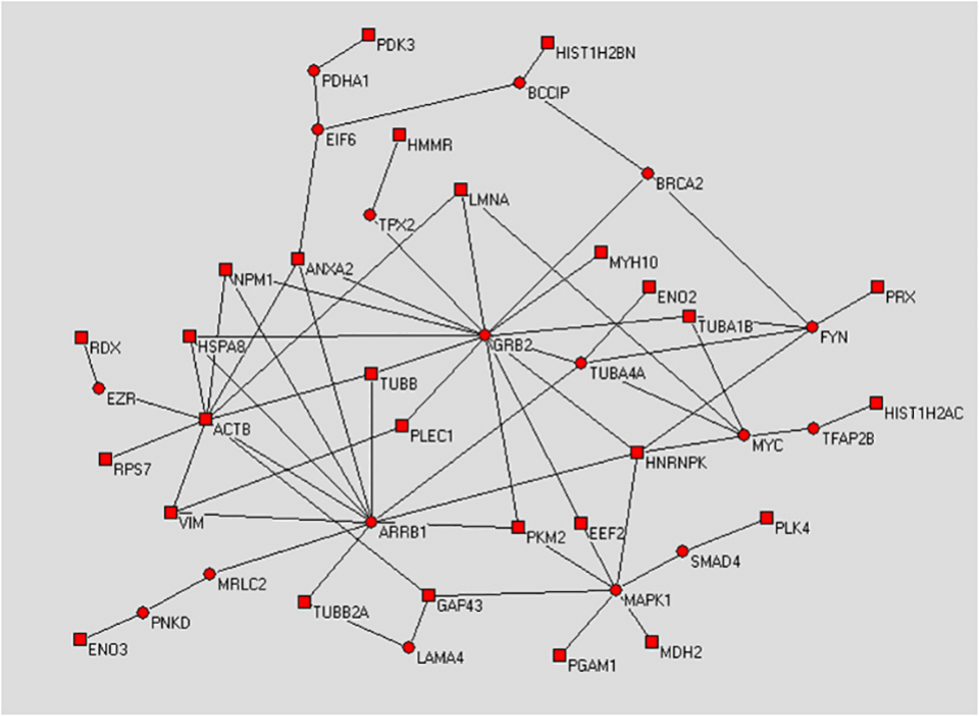
Constructed minimum protein-protein interaction network between targeted proteins of AGS-IV. Square nodes denote identified differentially expressed proteins of AGS-IV. Circular nodes are proteins that link the identified proteins together. The network was decomposed into topological compact modules by a simulated annealing algorithm. Symbols and full names of the intermediate partners in the network are shown in **Table 2**.

To understand the relationship among the putative targets of AGS-IV, we mapped the differentially expressed proteins onto the protein-protein interaction network of the human genome. We found that 27 of them can be linked into one sub-network either through direct interactions or with only one intermediate protein, suggesting that most of the targets are located in the neighborhood of each other in the human protein network (**Fig 5)**. The proximity of proteins in the interactome indicates that they share common functions. To identify the functions of this network between targets, the AGS-IV associated proteins are mapped onto KEGG. The mapping pathways showed that 27 genes appeared in a total of 8 pathways. Pathway enrichment analysis was performed to identify pathways significantly that were regulated by AGS-IV associated proteins, and the P-values were computed for each of the 8 pathways with AGS-IV associated proteins. The computations generated 8 pathways with P-values smaller than 0.01 (**Table 3**), and therefore these pathways were regarded as key pathways involved in the neuroprotective effect of AGS-IV against glutamate-induced neurotoxicity in PC12 cells.

**Table 3:** Lists of KEGG pathways that are significantly regulated by AGS-IV.

Definition	total genes	mapped genes	pathway category	Fisher-P value
MAPK signaling pathway	273	7	Signal transduction	1.12×10–5
Toll-like receptor signaling pathway	101	5	Immune system	0.0081
Cell adhesion molecules (CAMs)	154	4	Signaling molecules and interaction	0.0044
Glycerophospholipid metabolism	82	3	Energy metabolism	0.0062
Calcium signaling pathway	178	3	Signal transduction	0.0041
Glycine, serine and threonine metabolism	34	2	Energy metabolism	0.0062
Phosphatidylinositol signaling system	78	3	Signal transduction	0.0065
Metabolic pathways	1197	9	Energy metabolism	0.0081

**Fig 6:**
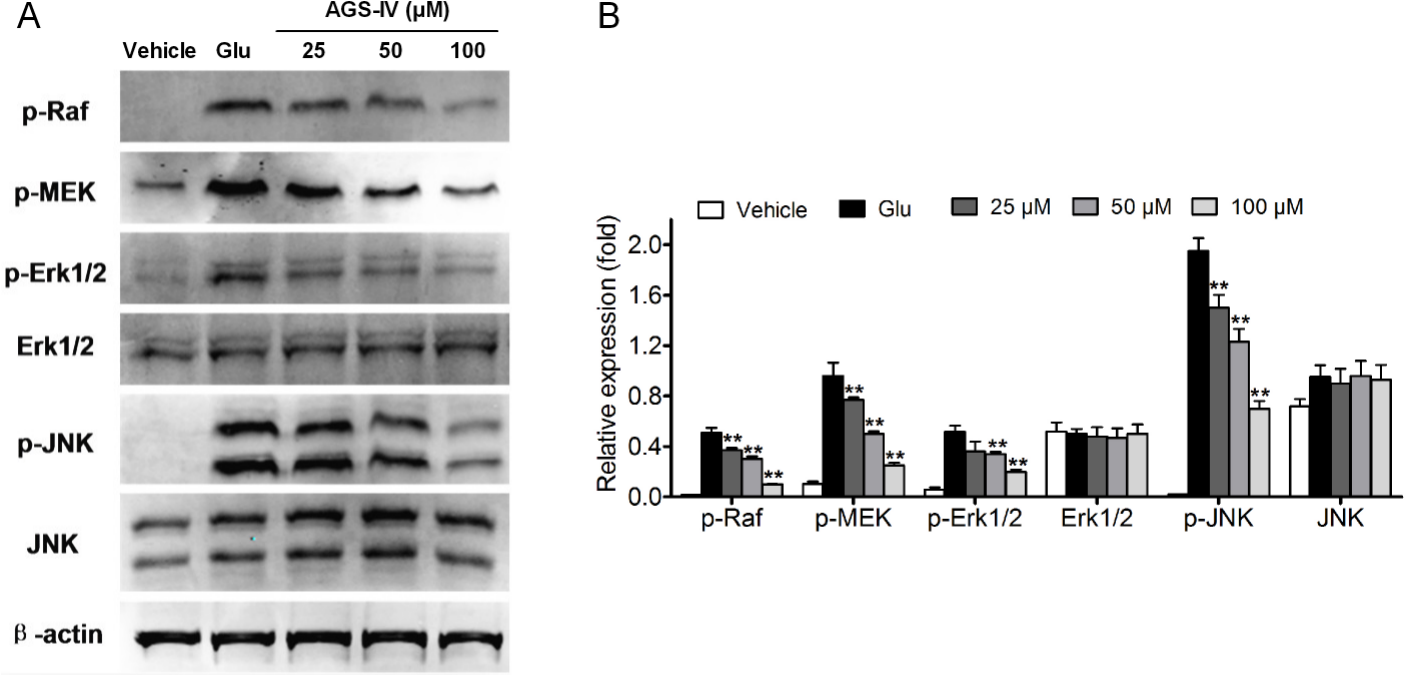
Involvement of Raf-MEK-ERK pathway in AGS-IV treated PC12 cells. PC12 cells were treated with different concentrations (25, 50 and 100 μM) of AGS-IV and then co-incubated with or without 5 mM glutamate (Glu) for 24 h. Data shown are the results of three different experiments and are represented as the relative densities of protein bands normalized to β-actin. Results are presented as means ± SEM of three assays. ** Significant difference compared with GLUs by one-way ANOVA analysis of variance with Tukey’s HSD post hoc test.

To validate the pathway enrichment result, we chosen MAPK signaling pathway for Western blot confirmation. Interestingly, MAPK1 (ERK1) was found as an intermediate protein in the protein-protein association network and it is possible that the ERK MAPK activation ultimately dictate whether glutamate induced neurotoxicity in PC12 cells. As shown in **Fig 6** and 5 mM glutamate exposure significantly increased p-Raf, p-MEK, p-ERK1/2, and p-JNK protein levels, which were inhibited by pretreated by AGS-IV in a dose-dependent manner. Therefore, Raf-MEK-ERK MAPK pathway was involved in the neuroprotective effect of AGS-IV against glutamate-induced neurotoxicity in PC12 cells.

## Discussion

Traditional Chinese medicine provides an extensive foundation for implementing a strategically focused pharmacological research program that aimed at the development of new drugs. *Astragalus mongholicus* Bunge, known as Huangqi in China, has been used as one of the primary Chinese tonic herbs for thousands of years. Recently there are some reports on the neuroprotective activities of the crude extract from Huangqi [34]. Screening active ingredients is an important step in the drug discovery of TCM. In this work, we find that AGS-IV, as a major active ingredient, is able to significantly attenuate neurotoxicity induced by glutamate in PC12 cells.

Glutamate is thought to be the major excitatory neurotransmitter working at a variety of excitatory synapses in the central nervous system [35]. It plays important roles in cellular process underlying synaptic plasticity, neuronal development and excitation via the activation of glutamate receptors [36,37]. Evidence is accumulating that high concentrations of glutamate and relative excitatory amino acid analogs cause a specific pattern of neurodegeneration in the brain of experiment animals, in primary culture of brain neurons and in some cultured neuron cell lines, including PC12 cell line, a rat pheochromocytoma cell line [38,39]. In high differentiated PC12 cells, lacking ionotropic glutamate receptors, the high concentration glutamate inhibits cystine uptake and depletes intracellular glutathione, which leads to the accumulation of reactive oxygen species (ROS) and ultimately causes cell death [40]. In the present study, we investigated whether the four cycloartane triterpenoid saponins were of neuroprotective activity in damaged PC12 induced by glutamate *in vitro*.

Target discovery plays an important role in clinical application of TCM. The proteomic approach is widely applied nowadays in understanding the molecular mechanisms of natural products and identification of new targets for therapeutics [41–43]. The 2D-nano-LC-MS/MS system applied is fully automated and displays advantages such as minimal loss of sample, no via contamination, and no sample dilution [33]. This study implemented the proteomics scheme to search globally for the differentially expressed proteins in PC12 cells affected by AGS-IV. In the present study, 13 proteins whose expressions were significantly changed under AGS-IV treatment were identified. In order to obtain an easier and non-biased interpretation of the results and to reduce the dimensionality of the multivariate data obtained from the LC-MS results, we analyzed the LC-MS chromatographic data using PCA. As an unsupervised method for pattern recognition, PCA multivariate data analysis procedure requires preprocessing of the raw data to generate a data matrix, the columns of which represent variables and the rows of which contain the samples that are included for analysis. These peak picking and peak alignment processes are important for creating a productive data matrix. After PCA processing, the AGS-IV treatment group (AGS-IVs) was clearly separated from the glumatate group (GLUs). Moreover, we constructed protein-protein association network, enriched the relevant pathways and verified that the Raf-MEK-ERK pathway was involved in the neuroprotective effect of astragaloside IV against glutamate-induced neurotoxicity in PC12 cells.

MAPK pathway plays a crucial role as transducers of extracellular stimuli into a series of intracellular phosphorylation cascades, which ultimately leads to cell differentiation, proliferation, survival or death [44,45]. To date, ERK MAPK, as one of major MAPK subfamilies, can be activated by inflammatory cytokines and extracellular stressors such as UV light, heat, and glumatate [46,47]. Activation of the Raf-MEK-ERK pathway has been shown to be a key regulator of neuronal apoptosis [48], which makes this pathway an important molecular target of neurodegenerative diseases therapy [49]. In the present study, pretreatment with AGS-IV dramatically inhibited the glumatate-induced increase in levels of phosphorylated Raf, MEK, and ERK MAPKs. These results indicate that AGS-IV prevents glumatate-induced apoptosis in PC12 cells by blocking the phosphorylation of Raf, MEK, and ERK MAPKs. In some instances, AGS-IV also modulates other hubs and exerts its influence on the others by acting on their surrounding neighbors. Previous studies on the effect of multi-target attacks in a network-based model suggested that weak inhibition of multiple targets could be more efficient than potent inhibition of a single hub target [50,51]. We can also see that proteins grouped together tend to be correlated in functionality or to participate in the same biological processes.

Overall, this network suggests that AGS-IV produce its beneficial effects on the neuroprotection against glutamate-induced neurotoxicity by several proteins associated with signal transduction, immune system, signaling molecules and interaction, and energy metabolism. Thus, the organization of the target protein interaction network exhibits a modular cooperative mode and may result in synergistic therapeutic action.

## Conclusions

The present study carried out a proteomics technique to search globally for the proteins after exposure of glutamate-treatment PC12 cells to AGS-IV, a purified component from TCM. 2D-nano-LC-MS/MS was conducted, and 40 differentially expressed proteins were found that might be target-related proteins of AGS-IV. Based on the results of comparative proteomics, we concluded that proteins associated with signal transduction, immune system, signaling molecules and interaction, and energy metabolism play important roles in neuroprotective effect of AGS-IV, although the precise roles of these identified molecules in neuroprotective effect of AGS-IV need further study. In addition, Raf-MEK-ERK pathway was involved in the neuroprotective effect of AGS-IV against glutamate-induced neurotoxicity in PC12 cells. The current study improves the understanding of mechanisms of AGS-IV neuroprotective effect, and provides prospects for the application of the comparative proteomics based on shotgun approach in biological mechanisms study.

In conclusion, the present results show that comparative proteomics based on shotgun approach is a valuable tool for molecular mechanism studies, since it allows the simultaneously evaluate the global proteins alterations.
